# Patterns of nucleotides that flank substitutions in human orthologous genes

**DOI:** 10.1186/1471-2164-11-416

**Published:** 2010-07-05

**Authors:** Lei Ma, Tingting Zhang, Zhuoran Huang, Xiaoqian Jiang, Shiheng Tao

**Affiliations:** 1 Bioinformatics Centre, Northwest A&F University, Yangling, Shaanxi, 712100, China; 2 College of Life Science, Northwest A&F University, Yangling, Shaanxi, 712100, China; 3 College of Life Science, Shihezi University, Shihezi, Xinjiang, 833200, China

## Abstract

**Background:**

Sequence context is an important aspect of base mutagenesis, and three-base periodicity is an intrinsic property of coding sequences. However, how three-base periodicity is influenced in the vicinity of substitutions is still unclear. The effect of context on mutagenesis should be revealed in the usage of nucleotides that flank substitutions. Relative entropy (also known as Kullback-Leibler divergence) is useful for finding unusual patterns in biological sequences.

**Results:**

Using relative entropy, we visualized the periodic patterns in the context of substitutions in human orthologous genes. Neighbouring patterns differed both among substitution categories and within a category that occurred at three codon positions. Transition tended to occur in periodic sequences relative to transversion. Periodic signals were stronger in a set of flanking sequences of substitutions that occurred at the third-codon positions than in those that occurred at the first- or second-codon positions. To determine how the three-base periodicity was affected near the substitution sites, we fitted a sine model to the values of the relative entropy. A sine of period equal to 3 is a good approximation for the three-base periodicity at sites not in close vicinity to some substitutions. These periods were interrupted near the substitution site and then reappeared away from substitutions. A comparative analysis between the native and codon-shuffled datasets suggested that the codon usage frequency was not the sole origin of the three-base periodicity, implying that the native order of codons also played an important role in this periodicity. Synonymous codon shuffling revealed that synonymous codon usage bias was one of the factors responsible for the observed three-base periodicity.

**Conclusions:**

Our results offer an efficient way to illustrate unusual periodic patterns in the context of substitutions and provide further insight into the origin of three-base periodicity. This periodicity is a result of the native codon order in the reading frame. The length of the period equal to 3 is caused by the usage bias of nucleotides in synonymous codons. The periodic features in nucleotides surrounding substitutions aid in further understanding genetic variation and nucleotide mutagenesis.

## Background

To understand the mechanisms underlying basic processes such as nucleotide mutation and fixation, DNA-protein interactions, DNA repair, and genetic diseases, it is important to study the influence of neighbouring nucleotides on substitutions in coding sequences [1-3]. Studies on single nucleotide polymorphisms (SNPs) have revealed large biases of nucleotide composition at the two immediately adjacent sites of SNPs [4]. Small biases can also occur farther away from polymorphic sites [4]. Analysis of the sequence context of SNPs has indicated that CpG dinucleotides dominate in polymorphic sites (SNPs) within non-CpG islands, whereas CpG dinucleotides are suppressed in polymorphic sites (SNPs) within CpG islands [5-7]. In plant chloroplast DNA, the mutation pattern correlates with the local base composition [8,9]. In a spectrum of 7,271 mutations in 574 disease-related genes, the surrounding sequences were found to exert only a local influence on mutations [10].

Periodic signals, specifically three-base periodicity, exist in prokaryotic and eukaryotic exons. Early explanations for three-base periodicity suggested a preference for some nucleotides in the first-codon positions of coding sequences, as well as an avoidance of some nucleotides in the second-codon positions [11]. Later research suggested that the periodicity in exons arose due to the bias of codon usage frequencies [12]. Other studies proposed that three-base periodicity was determined by a preference for triplets that are separated by 3, 6 and 9 nucleotides (3n distances) in mRNA [13,14]. As a gene monitoring approach, three-base periodicity can be used to predict protein coding sequences [15]. Intuitively, its variations should reveal coding frame shifts [16,17]. However, little is known regarding the effects of substitution and its context on three-base periodicity.

Detecting associations between substitutions and their contexts will depend on the size of the alphabet used and on the frequencies with which certain elements (monomers, dimers or n-mers over the chosen alphabet) occur along sequences. Thus, this type of approach has its limitations. For example, the arbitrary choices of oligomer length can miss information beyond the chosen window. Furthermore, for the chosen alphabet, the numerical value of compositional complexity will increase exponentially according to the arbitrary oligomer length. For example, the size of a 6-mer alphabet is 4^6 ^(4096) oligomers for DNA. In this sense, retrieving long oligomers in DNA becomes a computationally time-consuming task that requires very powerful computational facilities, which may not always be available.

A number of other processes can be useful for finding unusual patterns in biological sequences. One of these is the Kullback-Leibler divergence, a windowless technique that can reflect the usage bias of nucleotides in each column of a sequence alignment. In this study, we have used the Kullback-Leibler divergence and, based on relative entropy, we have been able to illustrate periodic patterns in sequences that flank one-base substitutions in human orthologous genes. Using a sine model to fit these periodic patterns, we could then assess how the known three-base periodicity was influenced in the vicinity of substitution sites. We discuss factors responsible for three-base periodicity, generated with the aid of two control sets in which codons were shuffled in two different manners.

In the present study, neighbouring-nucleotide patterns were found to differ among substitutions and transition was prone to occur in periodic sequences. We verified that a sine model of period equal to 3 was a good approximation for periodic signals at sites not in close vicinity of some substitutions. Overall, our findings support the conclusion that synonymous codon usage bias is responsible for three-base periodicity.

## Methods

### Sequences

We downloaded a dataset of 10,376 tri-alignments of human-chimpanzee-macaque orthologous genes from http://compgen.bscb.cornell.edu/orthologs/. This dataset [18] was derived from human genes in RefSeq [19], VEGA [20], and UCSC known Gene databases [21], and had been passed through a series of rigorous filters that would remove genes with annotation errors, sequencing and assembly errors, poor synteny relationships, incomplete alignments, frame-shifts, changes in exon-intron structure, and recent duplication evidences [18].

### Substitution inference method

The method used for inferring a nucleotide substitution in a tri-species alignment has been described previously [22,23]. For example, given that at a certain nucleotide site, the human gene has A and both the chimpanzee and macaque genes have G. We then can assume that the nucleotide G has changed to A in the human gene, i.e., G→A. There are 12 substitution categories, i.e., each type of nucleotide can change into any of the other three types. Based on the tri-alignments dataset, we inferred 36,459 substitutions and then excluded adjacent multiple-base substitutions, deletions and insertions. The remaining 35,561 substitutions were divided into 3 groups in terms of the codon positions at which substitutions occurred and were further divided into 12 categories according to substitution type (Additional file 1). Next, we extracted 100 nucleotides on each flank of each substitution and eliminated (if any) gaps in the sequences.

### Sequence alignment method

**Figure 1:**
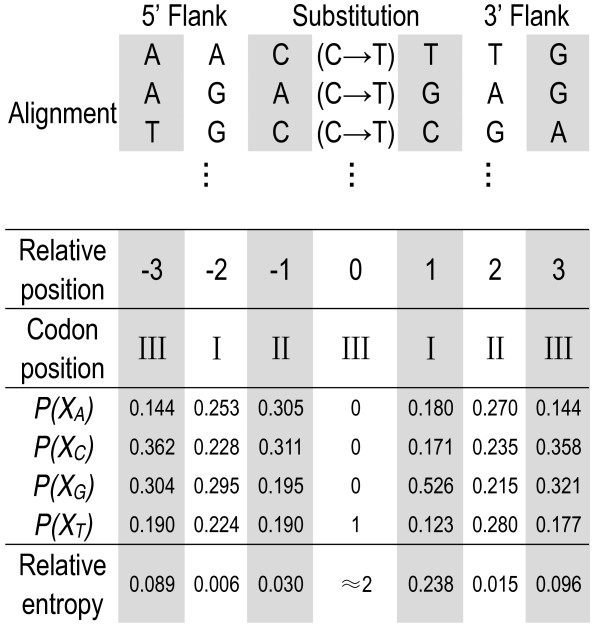
**An example of a sequence alignment and a relative entropy calculation**. This example shows the calculation of the relative entropies along the neighbouring-nucleotide positions of substitutions (C→T) that occurred at the third-codon positions. In an extracted sequence, the substitution position is labelled as 0, the 5' flank as negative and the 3' flank as positive. Extracted sequences are aligned by these corresponding positions so that the alignment is kept in the same reading frame. The frequencies *P*(*X*_*i*_) of four types of nucleotides for each column of this alignment are then used to calculate the relative entropy at each neighbouring-nucleotide position (see Equation 1). In this example, all nucleotides at site 0 (the substitution site) are T and the relative entropy approximates - *log*_2 _[*Q*(*X*_*T*_)]≈ 2 bits at this site.

We arbitrarily labelled the position of the substitution site as 'zero' in the extracted sequence; positions at the 5' flank were then designated as negative numbers and the positions at the 3' flank as positive numbers. We then aligned the extracted sequences by these corresponding positions (see Figure 1 for an example). We next calculated the frequencies of four nucleotide types in each column of that alignment. Lastly, we applied relative entropy to estimate the distance between the distribution of nucleotides at each neighbouring position and the background distribution (see Relative entropy below).

### Relative entropy calculation

Relative entropy at each position (or column) of a multiple sequence alignment followed the definition used in reference [24]:(1)

where *X*_*i *_is a random variable for A, C, G, or T, *P*(*X*_*i*_) represents the frequencies of four types of nucleotides in each column of a sequence alignment (see Figure 1 for an example), and *Q*(*X*_*i*_) is the background distribution of *X*_*i *_in sequences. The background frequencies for nucleotides A, C, G and T are 26.64%, 25.12%, 25.78% and 22.47% in human genes and 26.65%, 25.11%, 25.77% and 22.47% in chimpanzee genes. The relative entropy *H*(*P*||*Q*) represents a distance between the probability distributions *P *and *Q*. Relative entropy is always greater than zero, or equal to zero if and only if *P*(*X*_*i*_) = *Q*(*X*_*i*_) for all *i*. It is maximised when *P*(*X*_*i*_) = 1 for one *i *and the maximum is - *log*_2_[*Q*(*X*_*i*_)] ≈ 2 bits.

### Sine model application

We applied a sine model to fit periodic signals of relative entropies over nucleotide positions, as follows:(2)

where  is the predicted value of the relative entropy at nucleotide position *x*; *b*_*0 *_is the vertical shift, independent of the periodicity; *A*, the amplitude, is the peak deviation from the centre position; *ω*, the frequency, specifies how many nucleotide positions occur in an interval; and *φ*, the phase, specifies where the oscillation begins at *x *= 0. The entire waveform appears to be shifted in *x *by the amount *φ*. The peak is located at *x *= *ωn *+ *ω/4 - φ *if *A *is positive or *x *= *ωn - ω/4 - φ *if *A *is negative. We used SPSS statistical software (version 16.0, SPSS, Chicago IL, USA) to estimate these parameters for the model. We used the sum of squared residuals and the R^2 ^as criteria to examine the fit between the observed and predicted entropies.

### Estimation of P-values

It is instructive to estimate whether the probability of observing a particular entropy value is equal to or greater than chance. We randomly shuffled mononucleotides within each extracted sequence. We then realigned these shuffled sequences to calculate the relative entropy for each nucleotide position. This nucleotide-shuffling process, realignment and recalculation of relative entropy were repeated 1,000 times. The number of occurrences of these randomized relative entropies then became the P-value.

### Codon shuffling

The role of codon usage frequencies on periodical signals of relative entropy over neighbouring-nucleotide positions was examined by randomly shuffling codons within each extracted reading frame. Each codon-shuffled sequence strictly retained the codon usage frequencies of the corresponding native sequence. We then realigned these codon-shuffled sequences in the same reading frame and computed the relative entropy in each column of this alignment. This codon-shuffling process, realignment and recalculation were repeated 1,000 times to obtain the mean and standard deviation of the relative entropy in each position.

### Shuffling of synonymous codons

The role of synonymous codon usage on periodical signals of relative entropy was examined by randomly shuffling synonymous codons within a degenerate codon box in each extracted reading frame, without changing the position of any mononucleotide. In this way, the nature of protein sequences is totally preserved. We also realigned these shuffled sequences by their original nucleotide positions in the same reading frame, and then calculated the relative entropy in each column of this alignment. This shuffling, realignment and recalculation were repeated 1,000 times to obtain the mean and standard deviation of the relative entropy in each position.

### Multiple correspondence analysis

We applied the multiple correspondence analysis (MCA) of SPSS version 16.0 to study the relationship among gene GC content, substitutions and adjacent nucleotides. "Multiple correspondence analysis, also known as homogeneity analysis, quantifies nominal (categorical) data by assigning numerical values to the cases (objects) and categories, such that in the low-dimensional representation of the data, objects within the same category are close together and objects in different categories are far apart. Each object is as close as possible to the category points of categories that apply to the object. In this way, the categories divide the objects into homogeneous subgroups. Variables are considered homogeneous when they classify objects that are in the same categories into the same subgroups" (see SPSS version 16.0 manual [25]). Further detailed information regarding MCA algorithms can be obtained in [26].

MCA allows us to analyse the pattern of relationships of several categorical dependent variables. Each nominal variable comprises several categories in our study. As mentioned above, the variable *substitution *contains twelve categories, i.e., A→C, A→G, A→T, C→A, C→T, C→G, G→A, G→C, G →T, T→A, T→C and T→G. We then divided all genes involved in our study into six groups according to their GC contents: (1) GC% < 40%, (2) 40% <= GC% < 45%, (3) 45% <= GC% < 50%, (4) 50% <= GC% < 55%, (5) 55% <= GC% < 60%, and (6) GC% >= 60%. Thus, the variable *GC% *consists of six categories. Next, we built another two variables. One is the *5' adjacent base*, representing the 5' (-1) nucleotides immediately adjacent to substitutions; the other is the *3' adjacent base*, representing the 3' (+1) nucleotides immediately adjacent to substitutions. Each of these two variables contains four categories, i.e., A, C, G and T. In this way, a case (or object) in our study is to record one category of each of these four variables. We then constructed a table with cases as rows and variables as columns, according to the SPSS data format (Additional file 2).

## Results

### General patterns of flanking-nucleotides

**Figure 2:**
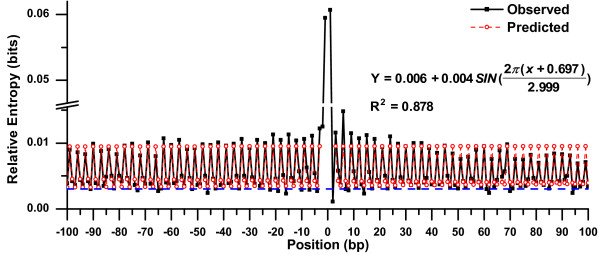
**Observed and predicted relative entropies over flanking nucleotide positions**. On the X-axis, the substitution site is labelled as 0, the 5' flank as negative and the 3' flank as positive. Equation 2 was employed to fit the relative entropies, given that the region between sites ±2 was excluded. The black solid squares represent observed relative entropies over neighbouring-nucleotide positions. The red circles represent the best-fit curve for the data. The horizontal reference line represents a threshold value. If the observed relative entropy is greater than or equal to the threshold value, its occurrence probability is lower than 0.001 by chance.

Using relative entropy, we presented an analysis of the statistical characteristics of the nucleotide usage at the neighbouring locations around substitutions in genes. To illustrate our method, Figure 1 shows an example for which relative entropy has been calculated. Figure 2 displays the relative entropies of 200 neighbouring-nucleotide positions around all substitutions, regardless of the reading frame. As substitutions are prevalent in the third-codon positions, Figure 2 primarily reflects the neighbouring effect of substitutions occurring at the third-codon positions. Additional file 3 illustrates three respective counterparts of substitutions that separately occurred at three codon positions. As mentioned in the Background, clear periodic signals of relative entropies were present in flanking sequences.

One of our aims is to assess how three-base periodicity was influenced in the vicinity of substitutions. We observed a trend that relative entropies of nucleotides adjacent to substitutions displayed a pronounced peak in the periodic spectrum. In other words, the usage of nucleotides surrounding the substitution site revealed bias. As shown in Figure 2, with increasing distance from the substitution site, the three-base periodicity improved in appearance and the substitution had less influence.

**Figure 3:**
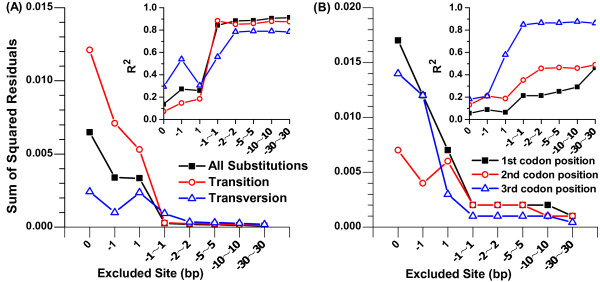
**Sine periodicity is influenced near the substitution site**. This figure reveals influence of substitution and its context on three-base periodicity. To assess the influence, we gradually excluded entropies at positions near the substitution site in attempt to improve the fitting of Equation 2. The main graph shows the sum of squared residuals and the inset graph shows the R^2^. X-axis represents the excluded neighbouring-nucleotide(s) in fitting process. (A) indicates groups of all substitutions (square), transition (circle) and transversion (triangle), while (B) indicates groups of substitutions which occurred at the first (square), second (circle) and third (triangle) codon positions.

To gain further insight, we employed a sine model (Equation 2) to fit periodic signals of relative entropies over neighbouring-nucleotide positions. Figure 3 reveals the fitting process whereby we gradually excluded entropies at positions near the substitution site in an attempt to improve the fit. In fact, Figure 3 indicates how long and to what degree three-base periodicity was influenced in the sequences flanking the substitution site. When excluding more positions near substitutions in the fitting process, the sum of squared residuals for Equation 2 decreased as an L-shaped curve, and the R^2 ^increased as an S-shaped curve (Figure 3). In addition, the points at site +1 and site -1 looked like "turning points", as can be seen in Figure 3. If the observed entropies at site +1 and site -1 were excluded from the fitting process, the sum of squared residuals dramatically decreased and the R^2 ^greatly increased (Figure 3). This implied that site ±1 did not belong to the three-base periodicity, whereas the observed entropies at positions away from site ±1 slightly affected the fitting. In this sense, the use of nucleotides at site ±1 was biased, so the entropies did not follow the sine periodicity.

### Periodic patterns in 12 substitution categories

**Figure 4:**
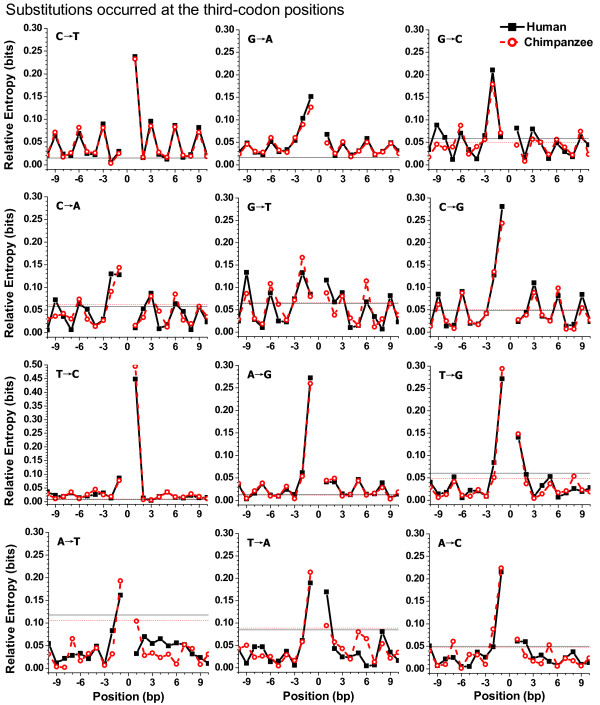
**Relative entropies of 12 substitution categories**. On the X-axis, the substitution site is labelled as 0, the 5' flank as negative and the 3' flank as positive. The horizontal reference line represents a threshold value. If the observed relative entropy is greater than or equal to the threshold value, its occurrence probability is lower than 0.001 by chance. Black solid lines (with squares) indicate results in human genes and red broken lines (with circles) indicate those in chimpanzee genes. To clarify fluctuations of small entropies, we did not show the entropy at site 0 (i.e., the substitution site), which approximates 2 bits (see methods).

It is instructive to survey the neighbouring effects on different substitutions. Figure 4 shows relative entropies across neighbouring-nucleotide positions of different substitutions that occurred at the third-codon positions. The corresponding results for substitutions that occurred at the first- and second-codon positions are displayed in Additional file 4. For the sake of clarity, Figure 4 and Additional file 4 only present the region from site -10 to site10. Outside this region, some categories still revealed the same periodicity shown in Figure 4 and Additional file 4. In addition, we analysed the neighbouring effect of 35,447 substitutions in chimpanzee orthologous genes. The patterns of flanking sequences were similar between human and chimpanzee genes for substitutions that occurred at the third-codon positions, but were different for some categories that occurred at the first-and second-codon positions.

In most cases, the highest entropy value was located at sites -1 or +1 (Figure 4 and Additional file 4). For example, site +1 loaded the highest entropy value of category C→T and site -1 loaded the highest one of category G→A. In cases of C→T and G→A, almost all entropy values were significant at the level of 0.001, as shown in Figure 4. Interestingly, in both human and chimpanzee genes, for category G→C that occurred at the third-codon positions, there was an exception whereby the highest entropy value was located at site -2 (Figure 4).

As seen in Figure 4, the category C→T, G→A, G→C, C→A, G→T and C→G, in which substitutions occurred at the third-codon positions, presented an apparent three-base periodicity, which peaked at the third-codon positions (3n) and bottomed at the first (3n-2) or second (3n-1) codon positions, neglecting the nucleotides adjacent to the substitution site. Entropies in categories T→C and A→G presented a weak period. In addition, entropies in the other four categories (T→G, A→T, T→A and A→C) were not significant at the level of 0.001, except for the adjacent nucleotides.

On the other hand, substitution categories that occurred at the first- or second- codon positions reflected weaker periodicity than that seen at the third positions (Additional file 4). At the level of 0.001, for almost all transversion categories that occurred at the first- and second-codon positions, entropies were not significant beyond the region from -2 to 2.

### Native and simulated periodicities

To survey the reasons that could underlie the three-base periodicity in flanking sequences of substitutions, we established two control sets in which codons were shuffled in two different manners. Table 1 and Additional file 5 show the parameters of Equation 2 for fitting entropies in the native and simulated datasets. Because of the irregularity of the relative entropies in the region from site -2 to +2, as mentioned above, we excluded this region when estimating parameters in the native and the synonymous-codon-shuffled datasets of shown in Table 1 and Additional file 5.

**Table 1:** Parameters estimated by Equation 2 for substitutions occurring at the third-codon positions.

Category	**Native**^**A**^				**Codon shuffling**^**B, D**^				**Synonymous codon shuffling**^**C, D**^			
			
	**R**^**2**^	*Peak Loc*.	*A*	*ω*	**R**^**2**^	*Peak Loc*.	*A*	*ω*	**R**^**2**^	*Peak Loc*.	*A*	*ω*
*All*	0.863	3n - 0.275	0.007	2.997	0.994	3n - 0.205	0.005	2.997	0.983	3n - 1.218	0.014	3.001
*TS*	0.844	3n - 0.312	0.007	2.998	0.997	3n - 0.255	0.006	2.998	0.979	3n - 1.203	0.015	3.000
*TV*	0.438	3n - 0.062	0.005	2.995	0.947	3n + 0.043	0.004	2.996	0.917	3n - 1.293	0.012	3.001
												
C→T	0.930	3n - 0.006	0.029	2.999	0.999	3n + 0.017	0.027	3.000	0.916	3n - 1.275	0.012	3.001
A→G	0.852	3n - 1.156	0.015	3.000	0.999	3n - 1.151	0.015	3.000	0.929	3n - 1.137	0.018	3.000
G→A	0.833	3n - 0.088	0.014	3.001	0.995	3n - 0.065	0.007	2.998	0.924	3n - 1.239	0.015	3.000
T→C	0.787	3n - 1.068	0.011	3.000	0.998	3n - 1.032	0.009	3.000	0.940	3n - 1.141	0.017	3.000
G→T	0.724	3n + 0.059	0.031	3.000	0.998	3n + 0.067	0.027	3.000	0.547	3n - 1.383	0.011	2.997
C→A	0.721	3n + 0.061	0.026	2.997	0.914	3n - 1.925	0.019	3.000	0.487	3n - 1.397	0.010	3.009
C→G	0.703	3n + 0.051	0.025	3.002	0.997	3n + 0.040	0.026	3.000	0.561	3n - 1.377	0.009	2.996
G→C	0.644	3n + 0.195	0.027	2.999	0.993	3n + 0.202	0.028	3.000	0.594	3n - 1.628	0.014	3.002
T→G	0.495	3n - 1.182	0.015	3.001	0.996	3n - 1.181	0.015	3.000	0.667	3n - 1.141	0.018	3.002
A→C	0.482	3n - 1.198	0.016	3.001	0.996	3n - 1.187	0.016	3.001	0.669	3n - 1.138	0.021	3.001
T→A	0.342	3n - 1.128	0.014	3.001	0.997	3n - 1.210	0.015	3.001	0.546	3n - 1.123	0.019	3.001
A→T	0.321	3n - 1.062	0.017	3.000	0.973	3n - 1.044	0.015	3.002	0.591	3n - 1.133	0.025	3.000

A: Parameters for fitting the periodic signals in the native flanking sequences, given that the region between site ±2 was excluded. B: Parameters for fitting the periodic signals in the corresponding codon-shuffled sequences. C: Parameters for fitting the periodic signals in the corresponding synonymous-codon-shuffled sequences, given that the region between site ±2 was excluded. D: Parameters were estimated from the mean for 1,000 independent random datasets. The following abbreviations and symbols are used in the table: *Peak Loc *represents peak location in the periodicity; *A *denotes amplitude; *3n *means the multiple of three, corresponding to the third-codon positions; *ω *denotes how many nucleotide sites occur in an interval; *All *represents all substitutions; *TS *and *TV *represent transition and transversion, respectively.

In the native dataset, at the threshold R^2 ^value of 0.7, Equation 2 is able to explain the three-base periodicity in 7 out of 12 substitution categories that occurred at the third-codon positions (Table 1), implying that Equation 2 was a good approximation for the three-base periodicity not in the vicinity of those substitutions. However, Equation 2 was not an ideal model for substitution categories that occurred at the first- and second-codon positions at the threshold R^2 ^value of 0.7 (Additional file 5). These may need a more complex model than is provided by Equation 2.

In the first control set, in order to assess the role of the codon usage frequencies on three-base periodicity in sequences, we shuffled codons within each extracted reading frame. The order of codons was the only difference between the native sequence and this artificial coding sequence. Additional file 6 shows the relative entropies of 21 nucleotide positions that were randomly chosen from this artificial dataset.

Interestingly, we found a trend whereby the codon-shuffled sequences revealed better periodicity than did the native sequences (Additional file 6 and Table 1). The sine model (Equation 2) was a perfect model to explain the three-base periodicity in the codon-shuffled dataset. In Table 1, all R^2 ^values for the codon-shuffled dataset were greater than 0.9. However, in the native dataset column of Table 1, only the R^2 ^of category C→T was greater than 0.9, and the R^2 ^of categories G→C, T→G, A→C, A→T, and T→A were lower than 0.7. Furthermore, this trend was also observed in the codon-shuffled dataset derived from substitutions that occurred at the first- and second-codon positions (Additional file 5). As seen in Additional file 5, at the threshold R^2 ^value of 0.7 for codon-shuffled dataset, Equation 2 was able to fit the sine periodicity in all 12 substitution categories that occurred at the first-codon positions and in 10 out of 12 categories that occurred at the second-codon positions.

**Figure 5:**
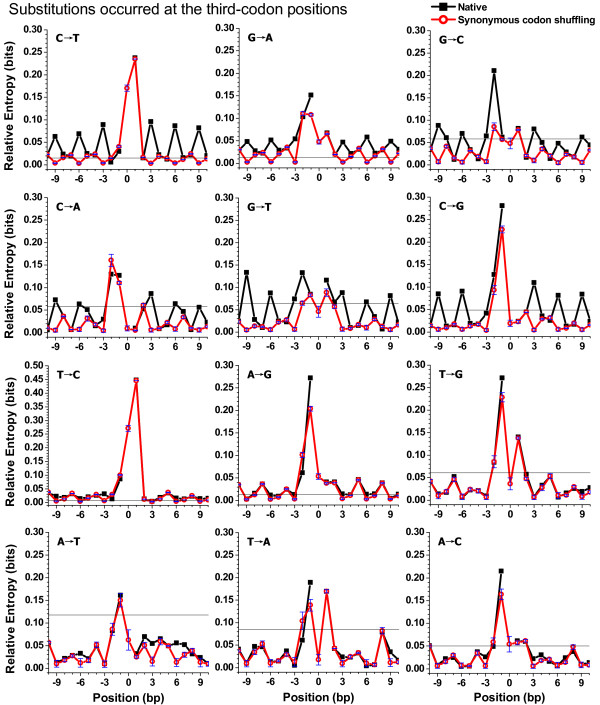
**Entropies in native and synonymous-codon-shuffled datasets**. On the X-axis, the substitution site is labelled as 0, the 5' flank as negative and the 3' flank as positive. The horizontal reference line represents a threshold value. If the observed relative entropy is greater than or equal to the threshold value, its occurrence probability is lower than 0.001 by chance. Means and standard deviations for 1,000 independently synonymous-codon-shuffled samples are indicated by the red circles and blue error bars.

In the second control condition, we tested the role of synonymous codon usage on three-base periodicity by only randomly exchanging synonymous codons within a degenerate codon box in each extracted reading frame. In this shuffling process, we did not change any position of mononucleotides and strictly kept the native protein sequences. Figure 5 and Additional file 7 compare relative entropies between the native and the synonymous-codon-shuffled dataset.

We found an apparent change in the relative entropies at the third-codon positions before and after synonymous codon shuffling. In Figure 5, for some categories (C→T, G→A, G→C, C→A, G→T, and C→G), the third-codon positions (3n) were the peak location in the native curves, but this changed to the trough location in the synonymous-codon-shuffled curves. This change was also observed in Table 1. For the six categories described above, the peak location in the native column of Table 1 approximated site 3n, but that in the synonymous-codon-shuffled column was near to site 3n-1. Further calculation revealed that the trough location in the synonymous-codon-shuffled dataset was closed to site 3n, which was the original peak location of the native dataset for the above six categories. This change implied that the use of nucleotides at the third-codon positions approximated a uniform after shuffling process. This trend was also revealed following comparison of the native dataset with the synonymous-codon-shuffled dataset for substitutions that occurred at the first- and second-codon positions (Additional file 7). However, as seen in Figure 5, entropies at the first- (3n-2) and second- (3n-1) codon positions changed only slightly before and after this kind shuffling.

On the other hand, the value of the relative entropy at site 0 (i.e., the substitution site) in the native dataset was certainly close to 2 bits (see methods), although this is not indicated in Figure 4 and 5. Interestingly, for substitution categories occurring at the third-codon positions, the entropy value at site 0 in the synonymous-codon-shuffled dataset was lower than 0.3 bits (Figure 5), implying the degeneracy at the third-codon positions. However, entropies approximated 2 bits at site 0 for substitutions that occurred at the second-codon positions in the synonymous-codon-shuffled dataset (Additional file 7-B), indicating that the second-codon positions were highly conserved.

### Biases of the adjacent nucleotides

The use of immediately adjacent nucleotides revealed differences both among substitution categories and within a category that occurred at three codon positions. For example, G at site +1 was the most frequent nucleotide for category C→T that occurred at the second- and third-codon positions, but T at +1 occurred the most frequently for C→T occurring at the first-codon positions. Table 2 shows the frequency of each nucleotide type at site ±1.

**Table 2:** Usage (%) of nucleotides adjacent to substitutions occurring at three codon positions.

Category	Site	1st					2nd					3rd				
				
		Entropy	A	C	G	T	Entropy	A	C	G	T	Entropy	A	C	G	T
C→T	-1	0.096	18.2	39.9	27.0	14.9	0.027	30.7	24.0	29.9	15.4	0.030	30.5	31.1	19.5	19.0
	1	0.323	7.3	22.4	21.2	49.2	0.159	16.2	21.5	47.0	15.3	0.238	18.0	17.1	52.6	12.3
G→A	-1	0.231	13.1	50.8	18.5	17.6	0.392	26.3	55.6	12.8	5.3	0.152	27.0	42.1	10.7	20.3
	1	0.095	20.1	27.2	16.3	36.3	0.050	15.7	29.4	30.2	24.7	0.068	18.0	26.1	38.1	17.8
A→G	-1	0.170	22.8	46.3	13.1	17.9	0.229	43.7	33.1	17.2	6.0	0.272	28.3	49.4	8.7	13.6
	1	0.232	14.2	24.0	15.1	46.7	0.100	15.4	20.8	27.4	36.5	0.041	19.0	22.0	34.4	24.6
T→C	-1	0.022	28.4	30.1	25.3	16.2	0.119	41.8	17.3	28.9	11.9	0.086	41.1	25.1	16.1	17.7
	1	0.408	9.2	21.4	13.9	55.4	0.164	15.6	15.5	46.2	22.6	0.448	15.2	11.6	63.2	10.1
A→C	-1	0.063	23.9	37.7	23.3	15.1	0.171	35.2	26.0	33.5	5.3	0.215	16.5	50.1	15.1	18.4
	1	0.088	33.1	12.5	34.4	20.0	0.019	30.0	21.6	30.4	18.1	0.061	27.9	19.2	37.4	15.6
A→T	-1	0.087	18.8	37.0	30.4	13.8	0.061	16.8	29.4	35.0	18.9	0.161	17.3	45.5	15.0	22.2
	1	0.235	10.9	39.9	13.8	35.5	0.229	7.0	27.3	25.9	39.9	0.032	20.4	21.5	33.6	24.5
C→A	-1	0.042	22.3	33.0	29.1	15.5	0.073	38.1	24.7	25.1	12.1	0.127	13.2	41.5	22.3	23.0
	1	0.043	21.8	27.4	34.7	16.0	0.083	20.9	39.8	17.6	21.8	0.010	30.5	27.1	22.7	19.8
C→G	-1	0.088	17.6	36.8	17.8	27.8	0.013	31.5	21.0	27.2	20.3	0.281	11.9	48.6	11.4	28.1
	1	0.141	25.4	33.2	9.5	31.9	0.161	16.1	37.4	13.1	33.4	0.024	23.6	28.3	20.2	27.9
G→C	-1	0.043	26.0	20.5	36.2	17.3	0.221	40.3	11.0	38.8	9.9	0.062	22.4	25.0	18.2	34.4
	1	0.092	39.4	18.3	15.7	26.6	0.039	21.3	32.7	19.8	26.2	0.081	21.6	22.3	40.7	15.3
G→T	-1	0.040	19.2	29.5	32.7	18.5	0.077	20.7	31.6	34.8	12.9	0.085	17.2	28.0	19.7	35.1
	1	0.407	7.5	55.9	12.1	24.6	0.181	11.6	43.9	29.0	15.5	0.116	14.1	37.5	32.4	16.0
T→A	-1	0.028	24.2	33.7	21.1	21.1	0.069	32.2	34.4	18.9	14.4	0.189	18.0	44.1	11.1	26.8
	1	0.269	8.3	46.9	16.7	28.1	0.153	10.0	23.3	40.0	26.7	0.170	19.6	19.2	48.1	13.1
T→G	-1	0.118	15.2	34.1	17.4	33.3	0.046	20.4	19.5	35.4	24.8	0.272	11.9	48.4	11.9	27.8
	1	0.497	3.0	51.5	12.1	33.3	0.221	9.7	19.5	47.8	23.0	0.141	16.6	18.7	45.9	18.9

### Relationships among gene GC%, substitutions and adjacent nucleotides

**Figure 6:**
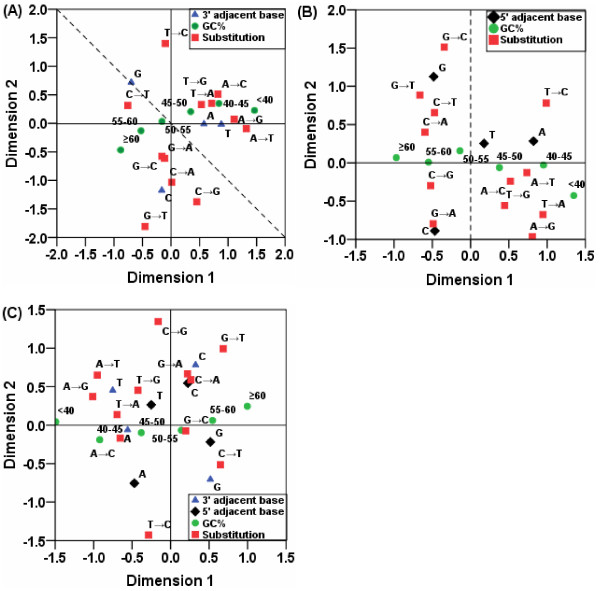
**Relationship among gene GC% groups, substitution categories and adjacent nucleotides**. The MCA of SPSS version 16.0 was used to analyse the relationship among gene GC content, substitutions and adjacent nucleotides. Figure (A) represents the analysis for the relationship among the 3' (+1) adjacent base, substitution categories and gene GC% groups. Figure (B) represents the analysis for the relationship among the 5' (-1) adjacent base, substitution categories and gene GC% groups. Figure (C) represents the analysis for the relationship among the 3' (+1) adjacent base, the 5' (-1) adjacent base, substitution categories and gene GC% groups.

We used the multiple correspondence analysis (MCA) of SPSS version 16.0 to analyse the relationships among four variables, i.e., gene GC% groups (*GC%*), *substitution*, the 5' (-1) immediately adjacent nucleotides (*5' adjacent base*) and 3' (+1) immediately adjacent nucleotides (*3' adjacent base*). Figure 6 illustrates the MCA on the four variables and Additional file 8 shows other output results from the MCA using SPSS version 16.0.

We then focused on interpreting the resulting MCA plots (Figure 6). The plots obtained by MCA graphically illustrate the underlying multi-dimensional relationships between categories and between variables [25]. The interpretation of the MCA plots indicates that closely located points are more correlated than are distantly located points. It is easy to see which categories of the same variable are similar to each other or which categories of different variables are related. For example, substitution G→A point is located near 5' (-1) adjacent base C point (Figure 6B), therefore, the dinucleotide substitutions CG→CA come into being as a mechanism of methylated CpGs, which subsequently change to TpGs or CpAs.

In MCA plots, the distance of a point from the origin reflects the difference from its "average" response pattern. The average response pattern corresponds to the most frequent category of each variable. In this context, the GC% categories of 45%-50% and 50%-55% were very close to the origin, indicating that they were not well differentiated in the two dimensions (Figure 6). This makes sense, since the average GC% of genes involved in the present study was 50.9%. In contrast, the farthest points from the origin reflected the highest variability or uncertainty in the distribution of their frequencies.

As categories of a variable are separated farther in a dimension, the categories begin to be better represented in that dimension. Focusing on dimension 1 (the horizontal axis) in Figure 6 (A, B, and C), the categories of the variable GC% were far apart. However, along dimension 2 (the vertical axis), the GC% categories were very close. Furthermore, the discrimination measure in Additional file 8 indicated the percentage of variance explained by a dimension. The discrimination measure is equal to a squared correlation coefficient of the quantified variables with scores on an axis [25]. Large discrimination measures correspond to a large spread among the categories of the variable and, consequently, indicate a high degree of discrimination between the categories of the variable along that dimension [25,27]. The value of discrimination measure for the GC% categories on dimension 1 was greater than that on dimension 2 (Additional file 8). These results indicated that dimension 1 correlated with the variable GC%.

On the other hand, the categories for substitutions were spread far apart along both dimensions, indicating that substitution categories were well discriminated in both dimensions. Figure 6B shows that the substitutions were divided into two groups by the vertical reference line. The substitutions where A or T changed to another nucleotide are located to the right side of the reference line, while the substitutions from G or C to another nucleotide are located to the left side of the reference line. Similarly, the substitutions in Figure 6A are also separated by a diagonal reference line running from top left to bottom right.

If the different categories of a variable are located in the same graphic region or in the same direction relative to the original point (0, 0) in the MCA plots, they will have similar properties. In the right upper quadrant of Figure 6A, substitutions (A→C, A→G, and T→G) are located in the same region. These substitutions have a shared property in that they all represent replacements of a "weak" A:T bond with a "strong" G:C bond.

If the categories from different variables are located in the same graphic region or the same direction relative to the original point (0, 0), they are associated with each other. In Figure 6A, some "weak-to-strong" substitutions (A→C, A→G, and T→G) and GC-poor categories (<40%, 40%-45% and 45%-50%) are distributed in the same region, which indicated that these "weak-to-strong" substitutions seemed to be associated with these GC-poor categories. Similarly, substitution A→C and A→T are also close to these GC-poor categories, as shown in Figure 6B. In addition, these "weak-to-strong" substitutions are near the nucleotides A and T, suggesting that these substitutions may prefer base A or T as neighbours (Figure 6A and 6B).

Furthermore, we performed MCA on data including both the 3' (+1) and 5' (-1) adjacent bases, as shown in Figure 6C. Relationships seen in Figure 6C between categories and between variables are similar to those seen in Figures 6A and 6B which show analysis on data including either the 3' (+1) or 5' (-1) adjacent base. For example, the association of some "weak-to-strong" substitutions with the GC-poor categories also can be seen in Figure 6C. Moreover, both the 3' (+1) and 5' (-1) adjacent bases are spread farther apart in Figure 6C than in Figures 6A and 6B. In addition, the 3' (+1) adjacent base C and the 5' (-1) adjacent base C are located in the same direction relative to the original point (0, 0), implying probable correlation. However, the 3' (+1) adjacent base A is far away from the 5' (-1) adjacent base A.

The MCA results confirm the previous view that a GC-poor sequence may have more likelihood of nucleotide substitutions from A or T to another base [23]. The "weak-to-strong" substitutions may be influenced by the immediately adjacent nucleotide A or T. These association could potentially result from variations in the pattern of substitution, from localised selection for increased GC content, or from biased gene conversion (BGC), which would favour fixation of "weak-to-strong" substitutions [28,29].

## Discussion

The aim of the present study was to uncover the patterns of nucleotides that flank one-base substitutions in human orthologous genes, in order to estimate how three-base periodicity might be influenced in the sequences that flank substitutions. We used relative entropy as a tool to characterise the use of nucleotides that flank substitutions. We then fitted a sine model to the relative entropy values and verified that a period-three sine is a good approximation for periods at sites not in the close vicinity of some substitution categories.

### Influence of substitution and its context on three-base periodicity

The present investigations suggested that the known three-base periodicity was affected in the context of substitutions. The periodic patterns of flanking nucleotides varied both among substitution categories and within a category that occurred at three codon positions. Periodic signals in transversion categories cannot be explained by a sine model (Equation 2) at the R^2 ^level of 0.7, indicating that transversion may be prone to occur in coding sequences with a weak periodicity. In contrast, the sine model could explain the periodic behaviour in transition categories that occurred at the third-codon positions, implying that transition at the third-codon positions tended to take place in periodic sequences. In addition, in categories C→T and G→A, the nucleotide G prevailed in the first positions of some codons but did not do so in the second positions of some codons (data not shown). This finding was highly consistent with the RNY model (R = purine, Y = pyrimidine and N = purine or pyrimidine)[11]. Therefore, it is reasonable to think that substitution and its context influences the three-base periodicity in genes.

A link probably appeared between periodicity interruption and substitutions. The most common situation was one in which the periodicity was greatly affected in the close vicinity of a substitution. The periodic signals were interrupted near the substitution sites and they would then reappear away from the substitution sites. The usage biases of the flanking nucleotides extend no farther than two nucleotides from the substitution sites, similar to Krawczak's study [10]. These results add a further component for the study of substitutions in genes.

### Codon usage frequency is not the only origin of three-base periodicity

We also looked for the origin of three-base periodicity around substitutions. To determine the contribution of codon usage frequencies to three-base periodicity, we shuffled each extracted reading frame at the codon level. The shuffled sequence kept a strict codon usage frequency in the corresponding native sequence. Thus, if periodic signals of the native flanking sequences were to be determined by their codon usage frequencies alone, codon shuffling would not lead to changes in the periodic signals, and we would also see no difference between the native and the shuffled sequences. However, changes clearly appeared (Table1). Whether the native sequences revealed a periodic signal or not, their corresponding codon-shuffled sequences were reflected in the three-base periodicity (Table 1). For example, in the transversion categories, the native dataset presented weak periodicity but its codon-shuffled set showed strong periodicity (Table 1).

Differences between native and simulated sequences have also been reported in other studies [14]. However, these differences did not agree well with a previous suggestion [12] that codon usage frequencies should be the source of DNA periodicity in exons. This disagreement probably arose because the previous study emphasised entire coding sequences, similarly to Figure 2. In comparison, our study concentrated on the sequence context of different substitutions. Therefore, differences appeared when the individual substitution categories were probed. We could not explain the three-base periodicity by the codon usage frequencies alone.

### Codon order determines the differences between the native and codon-shuffled datasets

The question arises, therefore, regarding the cause of the difference between the native and codon-shuffled datasets. The only difference between a native sequence and its corresponding codon-shuffled sequence is the codon order seen in the reading frame. In this case, random changes in the native codon order lead to differences between the native and shuffled sequences. The codon order of genes is associated with mRNA secondary structure, encoded protein, DNA-protein interaction and other biological characteristics. In addition, the triplets in mRNA that are spaced in 3n distances can produce three-base periodicity [13], indicating that certain codon combinations probably result in the periodicity in exons [14]. Therefore, the order of codons in the native sequences determines their differences from the codon-shuffled sequences, and also contributes to differences in neighbouring effects among substitution categories.

On the other hand, clearly periodic signals of the relative entropies tended to appear in the codon-shuffled sequences, even if these signals could not be identified in the corresponding native sequences (Table 1). A similar study shows that simulated coding sequences, which were created using codon usage frequencies only, demonstrate DNA periodicity very similar to the observed in real exons [12]. However, another study suggested that three-base periodicity in biological sequences had specific characteristics not reproduced in computer-generated exon-like sequences based on codon usage frequencies [14]. This discrepancy may have arisen due to differences in methodology. The previous study [14] found that some triplets in mRNA appeared several times at a fixed distance (e.g. 3n) without interruption, so that triplets provided a good periodicity. The method used in that study was very useful in detecting triplets spaced by 3n distances in mRNA. For example, TTG triplets were found separated by 3, 3 and 6 nucleotides in that study. However, if we do not consider whether a triplet corresponds to a codon box; for instance, if we assume that TTG can occupy two codon boxes (e.g., TT in the first codon box and G in the following box), then codon-shuffling will certainly destroy this type of triplet spacing at a 3n-fixed distance in the native sequence. Consequently, the triplet periodic signals would disappear.

As mentioned in the Background, the relative entropy is a windowless technique that does not depend on oligonucleotides. Thus, the relative entropy technique uses an entirely different process to find periodic signals compared to the triplet technique. Furthermore, codon shuffling is prone to homogenise periodicities because it breaks the native cluster of codons in genes and makes codons uniform in a codon-column of sequence alignment. In this sense, periodicity in the codon-shuffled sequence reflects the usage bias of nucleotides among three codon positions.

Thus, our results confirm that the three-base periodicity is a result of certain codon clusters within coding sequences. The length of the period equal to 3 is caused by the usage bias of nucleotides within the genetic code.

### Synonymous codon usage bias is responsible for the three-base periodicity

The synonymous-codon shuffling introduced associations of codon degeneracy with three-base periodicity. Synonymous codons usually differ by nucleotides in the third-codon positions, while a few synonymous codons (6-fold degenerate) differ in the first- or second-codon positions. Thus, if we randomly changed synonymous codons in a degenerate codon box, we primarily caused random changes in the nucleotides at the third-codon positions. After synonymous codon shuffling, the use of nucleotides at the third-codon positions was close to uniform, resulting in loss of bias in the native sequences. In addition, by only varying two types of nucleotides in the first- or second-codon positions, the 6-fold degenerate codon groups could differ from each other. Therefore, entropy values at the third-codon positions in Figure 5, especially at site 0 (the substitution site), greatly decreased, whereas the values at the first- and second-codon positions changed only slightly. In other words, the bias of synonymous codon usage existed within the native coding sequences. The comparison between the native and synonymous-codon-shuffled dataset indicated that the usage bias of synonymous codons could account for the three-base periodicity in genes.

Synonymous codons are not randomly used both between genomes and among genes within a genome [27]. As mentioned in reference [30], many reasons underlie synonymous codon usage bias in coding sequences, including " (i) diversity in the (G+C)% at the third codon position [31], (ii) abundance of t-RNA molecules [32], (iii) overall base composition of genes [33], (iv) differences in the expression level of the genes [34], (v) differences in the cellular location of the genes in the genome [35], (vi) optimal growth temperature [36-38] and (vii) protein secondary structures [39,40]" [30].

### Limitations in flanking nucleotide length

Our study had one clear limitation. If a substitution was located near one end of the coding sequence, insufficient neighbouring nucleotides were available to study the periodic signals. Thus, we only examined 100 nucleotides flanking each side of a substitution. In total, 91.99% of the available substitutions had 100 or more nucleotides on the 5' flank, while 92.25% of substitutions had 100 or more nucleotides on the 3' flank. Nevertheless, this length setup did not influence the analysis of neighbouring effects near substitutions. In addition, some neighbouring effects agreed with what had been previously reported about the nucleotide biases over 300 nucleotides flanking SNPs [4].

## Conclusions

This study provides a useful way to illustrate unusual periodic patterns in the context of substitutions and verifies that a sine model of period equal to 3 is a good approximation for the three-base periodicity at sites not in the close vicinity of some substitutions. Our results offer further insight into the origin of the three-base periodicity. We confirm that the three-base periodicity is a result of certain codon clusters within coding sequences [14]. The length of the period equal to 3 is caused by the usage bias of nucleotides within synonymous codons. Our investigation of the influence of substitution and its context on the three-base periodicity in genes may be of great interest in analysing human genetic variation, as well as in designing gene predicting tools based on the principle of three-base periodicity.

## Authors' contributions

LM has made contributions to conception, analysis and interpretation of data, and drafted the manuscript. TZ involved in revising the manuscript critically for important intellectual content and performed statistical analysis. ZH and XJ proposed many additional suggestions for improving performance of computer programs. ST conceived the study, participated in its design and supported the research. All authors read and approved the final manuscript.

## Supplementary Material

Additional file 1 **Frequency of substitutions distributed in three respective codon positions**. This file shows the sample size of each substitution category that occurred at three codon positions. Click here for file

Additional file 2 **Data for MCA**. A table with cases as rows and variables as columns according to the SPSS data format. Click here for file

Additional file 3 **Counterparts of Figure **2**: Relative entropies in the flanking sequences of substitutions that separately occurred at three codon positions**. This file illustrates three respective counterparts for substitutions that separately occurred at three codon positions. The figure legend refers to Figure 2. Click here for file

Additional file 4 **Counterparts of Figure **4**: Relative entropies for 12 substitution categories that occurred at the first- and second-codon positions**. This file displays the corresponding results for 12 substitution categories that occurred at the first (A) and second (B) codon positions. The figure legend refers to Figure 4. Click here for file

Additional file 5 **Counterparts of Table **1**: Parameters estimated by Equation 2 for substitution categories that occurred at the first- and second-codon positions**. This file shows parameters estimated by Equation 2 for substitution categories that occurred at the first (A) and second (B) codon positions. Click here for file

Additional file 6 **Relative entropies of the nucleotide- and codon-shuffled datasets**. This file illustrates relative entropies across 21 nucleotide positions that were randomly chosen from nucleotide- and codon-shuffled sequences. The figure legend refers to Figure 4 except for labels on the X-axis. Points and error bars in the figures represent means and standard deviations for 1,000 independently random samples. Click here for file

Additional file 7 **Counterparts of Figure **5**: Entropies in native and synonymous-codon-shuffled datasets for substitutions that occurred at the first (A) and second (B) codon positions**. This file illustrates relative entropies in the native and synonymous-codon-shuffled dataset for substitutions that occurred at the first (A) and second (B) codon positions. The figure legend refers to Figure 5. Click here for file

Additional file 8 **Output of SPSS MCA**. This file illustrates the output results from the MCA of SPSS 16.0. Click here for file
