# Supplementary material for: Patterns of nucleotides that flank substitutions in human orthologous genes
Source: BMC Genomics. 2010 Jul 5;11:416. doi: 10.1186/1471-2164-11-416 (PMC2996944; doi:10.1186/1471-2164-11-416)
Supplement: Additional file 1 — Frequency of substitutions distributed in three respective codon positions. This file shows the sample size of each substitution category that occurred at three codon positions. [file 1471-2164-11-416-S1.PDF]

Frequency of substitutions distributed in three codon positions

Category	Codon Position			Total
	1st	2nd	3rd	
A→C	305	227	425	957
A→G	1417	1282	2896	5595
A→T	138	143	266	547
C→A	412	239	627	1278
C→G	370	305	875	1550
C→T	1256	1412	6590	9258
G→A	1916	1317	4801	8034
G→C	312	263	687	1262
G→T	281	155	610	1046
T→A	96	90	261	447
T→C	574	729	3603	4906
T→G	132	113	436	681
Transition	5163	4740	17890	27793
Transversion	2046	1535	4187	7768
Total	7209	6275	22077	35561
