# Supplementary material for: Patterns of nucleotides that flank substitutions in human orthologous genes
Source: BMC Genomics. 2010 Jul 5;11:416. doi: 10.1186/1471-2164-11-416 (PMC2996944; doi:10.1186/1471-2164-11-416)
Supplement: Additional file 3 — Counterparts of Figure 2: Relative entropies in the flanking sequences of substitutions that separately occurred at three codon positions. This file illustrates three respective counterparts for substitutions that separately occurred at three codon positions. The figure legend refers to Figure 2. [file 1471-2164-11-416-S3.PDF]

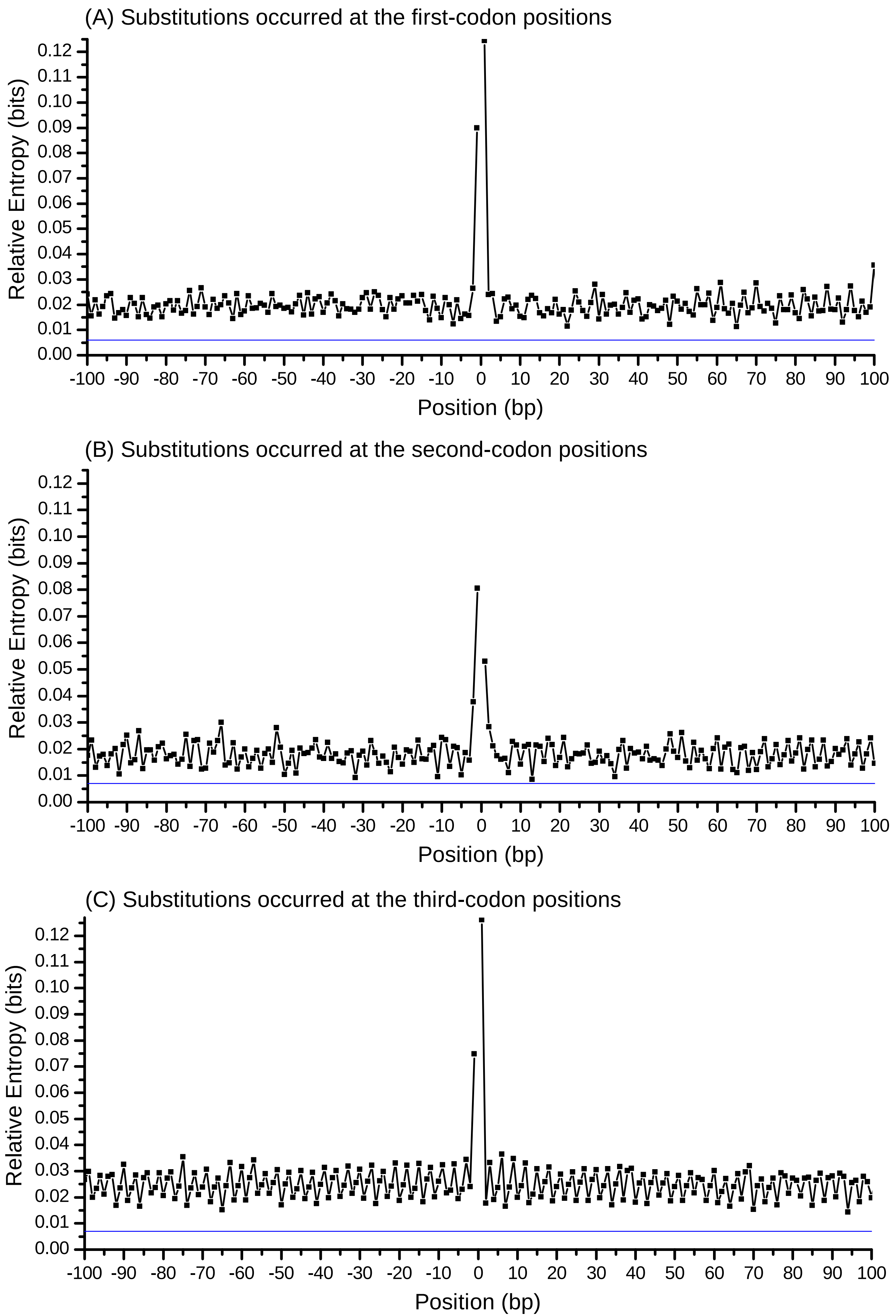
(A) Substitutions occurred at the first-codon positions
Relative Entropy (bits)
0.00
0.01
0.02
0.03
0.04
0.05
0.06
0.07
0.08
0.09
0.10
0.11
0.12
-100 -90 -80 -70 -60 -50 -40 -30 -20 -10 0 10 20 30 40 50 60 70 80 90 100
Position (bp)
(B) Substitutions occurred at the second-codon positions
Relative Entropy (bits)
Position (bp)
(C) Substitutions occurred at the third-codon positions
Relative Entropy (bits)
Position (bp)
