# Supplementary material for: Patterns of nucleotides that flank substitutions in human orthologous genes
Source: BMC Genomics. 2010 Jul 5;11:416. doi: 10.1186/1471-2164-11-416 (PMC2996944; doi:10.1186/1471-2164-11-416)

(A) Substitutions occurred at the first-codon positions

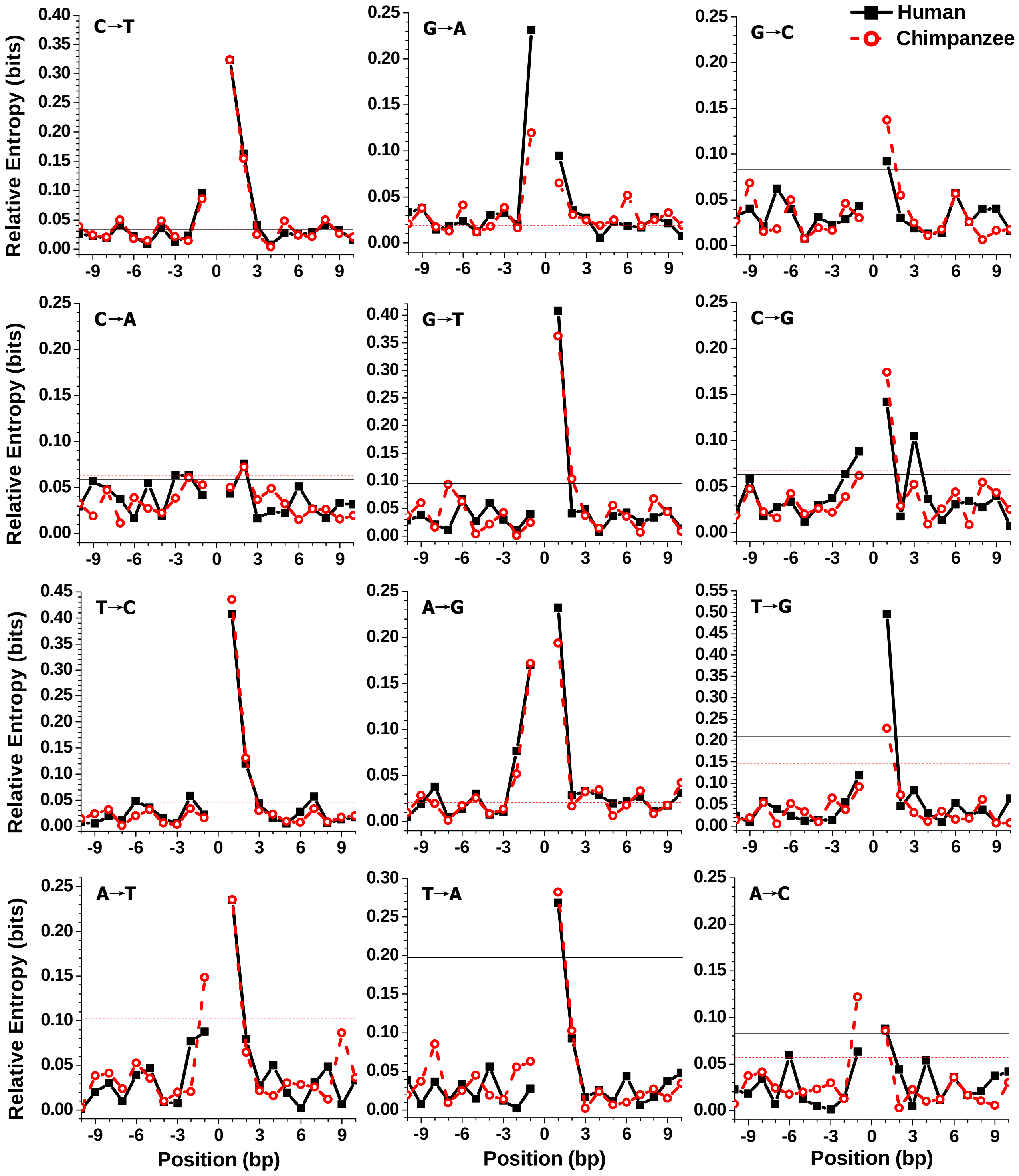

(B) Substitutions occurred at the second-codon positions

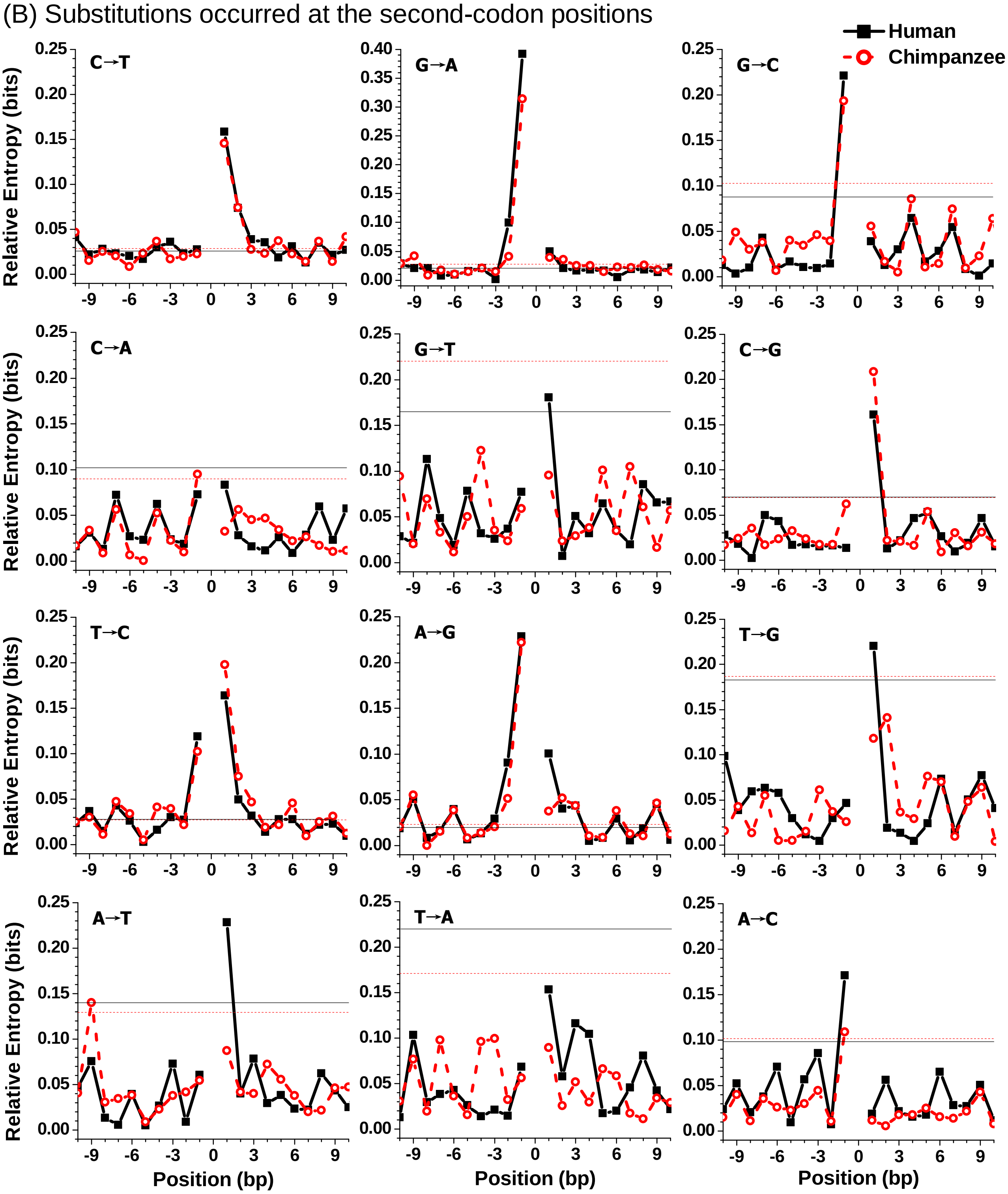

Supplement: Additional file 4 — Counterparts of Figure 4: Relative entropies for 12 substitution categories that occurred at the first- and second-codon positions. This file displays the corresponding results for 12 substitution categories that occurred at the first (A) and second (B) codon positions. The figure legend refers to Figure 4. [file 1471-2164-11-416-S4.PDF]
