# Supplementary material for: Patterns of nucleotides that flank substitutions in human orthologous genes
Source: BMC Genomics. 2010 Jul 5;11:416. doi: 10.1186/1471-2164-11-416 (PMC2996944; doi:10.1186/1471-2164-11-416)
Supplement: Additional file 5 — Counterparts of Table 1: Parameters estimated by Equation 2 for substitution categories that occurred at the first- and second-codon positions. This file shows parameters estimated by Equation 2 for substitution categories that occurred at the first (A) and second (B) codon positions. [file 1471-2164-11-416-S5.PDF]

Table (A) - Parameters estimated by Equation 2 for substitution categories occurring at the first-codon positions.

Category	Native [A]				Codon shuffling [B, D]				Synonymous codon shuffling [C, D]			
	R^2	*Peak Loc.*	*A*	ω	R^2	*Peak Loc.*	*A*	ω	R^2	*Peak Loc.*	*A*	ω
All	0.241	3n - 1.993	0.003	2.998	0.914	3n+0.105	0.002	2.994	0.923	3n - 2.255	0.012	3.000
TS	0.197	3n - 1.775	0.003	2.993	0.958	3n+0.309	0.003	2.993	0.906	3n - 2.245	0.012	3.000
TV	0.258	3n - 2.295	0.004	3.003	0.928	3n - 0.299	0.004	3.000	0.813	3n - 2.279	0.012	3.000
A→G	0.634	3n - 2.103	0.010	2.999	0.997	3n - 0.229	0.010	3.000	0.860	3n - 2.120	0.015	3.000
C→T	0.452	3n - 0.909	0.010	2.998	0.991	3n - 2.059	0.013	3.000	0.682	3n - 2.355	0.011	3.001
T→C	0.446	3n - 2.112	0.014	3.001	0.991	3n - 0.087	0.014	2.999	0.630	3n - 2.132	0.017	3.001
G→A	0.258	3n - 0.782	0.005	3.003	0.967	3n - 1.826	0.006	3.000	0.739	3n - 2.389	0.011	3.000
A→C	0.218	3n - 2.229	0.010	3.002	0.981	3n - 0.211	0.010	3.001	0.421	3n - 2.184	0.014	3.000
T→G	0.149	3n - 2.090	0.014	2.996	0.983	3n - 0.110	0.014	3.001	0.291	3n - 2.099	0.019	3.000
A→T	0.145	3n - 2.255	0.014	3.002	0.968	3n - 0.046	0.014	3.000	0.219	3n - 2.206	0.015	2.999
G→T	0.111	3n - 0.560	0.009	2.990	0.971	3n - 1.818	0.008	3.001	0.265	3n+0.400	0.011	3.004
G→C	0.091	3n - 0.436	0.007	3.008	0.978	3n - 1.393	0.007	3.003	0.331	3n+0.450	0.012	2.998
T→A	0.088	3n - 2.010	0.011	3.002	0.833	3n - 0.132	0.009	2.999	0.206	3n - 2.036	0.014	3.000
C→G	0.031	3n - 2.492	0.003	3.001	0.936	3n - 0.707	0.007	3.005	0.393	3n - 2.366	0.013	3.000
C→A	0.017	3n - 1.861	0.003	3.007	0.761	3n - 2.416	0.002	2.979	0.451	3n - 2.247	0.016	3.001

A: Native dataset, given that the region between site ± 2 was excluded.

B: Codon-shuffled dataset.

C: Synonymous-codon-shuffled dataset, given that the region between site ± 2 was excluded.

D: Parameters were estimated from the mean for 1000 independent random datasets.

The following abbreviations and symbols are used in the table: *Peak Loc* represents peak location in the periodicity; *A* denotes amplitude; *3n* means the multiple of three, corresponding to the first-codon positions; ω denotes how many nucleotide sites occur in an interval; *All* represents all substitutions; *TS* and *TV* represent transition and transversion, respectively

Table (B) - Parameters estimated by Equation 2 for substitution categories occurring at the second-codon positions.

Category	Native [A]				Codon shuffling [B, D]				Synonymous codon shuffling [C, D]			
	R^2	*Peak Loc.*	*A*	ω	R^2	*Peak Loc.*	*A*	ω	R^2	*Peak Loc.*	*A*	ω
All	0.497	3n - 0.329	0.004	3.003	0.899	3n - 0.441	0.003	3.002	0.927	3n - 0.272	0.012	3.001
TS	0.462	3n - 0.315	0.004	3.004	0.932	3n - 0.374	0.004	3.001	0.900	3n - 0.261	0.012	3.001
TV	0.128	3n - 0.392	0.003	2.997	0.773	3n - 0.827	0.003	3.008	0.748	3n - 0.312	0.011	2.999
A→G	0.651	3n - 0.235	0.014	3.002	0.989	3n - 0.378	0.009	3.000	0.788	3n - 0.172	0.018	3.001
T→C	0.399	3n - 0.253	0.009	3.005	0.990	3n - 0.261	0.009	3.000	0.684	3n - 0.189	0.014	3.002
A→C	0.319	3n - 0.244	0.016	2.998	0.968	3n - 0.309	0.016	3.001	0.455	3n - 0.179	0.019	3.000
G→T	0.276	3n - 1.775	0.018	3.002	0.969	3n - 1.752	0.019	3.000	0.153	3n - 0.890	0.008	2.997
A→T	0.213	3n - 0.133	0.019	3.006	0.988	3n - 0.155	0.019	3.000	0.325	3n - 0.136	0.023	3.003
G→C	0.148	3n - 1.500	0.009	3.003	0.911	3n - 1.670	0.009	3.006	0.198	3n - 0.721	0.009	2.995
G→A	0.099	3n - 2.002	0.003	2.992	0.871	3n - 2.029	0.003	2.991	0.636	3n - 0.311	0.010	3.001
T→G	0.065	3n - 0.059	0.010	2.992	0.595	3n - 0.154	0.003	2.999	0.210	3n - 0.134	0.016	2.994
T→A	0.051	3n+0.008	0.010	3.007	0.882	3n - 0.056	0.011	3.001	0.170	3n - 0.155	0.015	3.000
C→G	0.038	3n - 0.346	0.004	3.002	0.805	3n - 1.328	0.002	3.011	0.354	3n - 0.263	0.011	3.002
C→T	0.030	3n - 1.226	0.002	2.990	0.824	3n - 1.696	0.001	3.016	0.724	3n - 0.359	0.012	3.001
C→A	0.005	3n - 2.248	0.002	2.962	0.581	3n - 0.221	0.002	3.000	0.250	3n - 0.369	0.010	3.001

A: Native dataset, given that the region between site ± 2 was excluded.

B: Codon-shuffled dataset.

C: Synonymous-codon-shuffled dataset, given that the region between site ± 2 was excluded.

D: Parameters were estimated from the mean for 1000 independent random datasets.

The following abbreviations and symbols are used in the table: *Peak Loc* represents peak location in the periodicity; *A* denotes amplitude; *3n* means the multiple of three, corresponding to the second-codon positions; ω denotes how many nucleotide sites occur in an interval; *All* represents all substitutions; *TS* and *TV* represent transition and transversion, respectively
