# Supplementary material for: Patterns of nucleotides that flank substitutions in human orthologous genes
Source: BMC Genomics. 2010 Jul 5;11:416. doi: 10.1186/1471-2164-11-416 (PMC2996944; doi:10.1186/1471-2164-11-416)

(A) Substitutions occurred at the first-codon positions

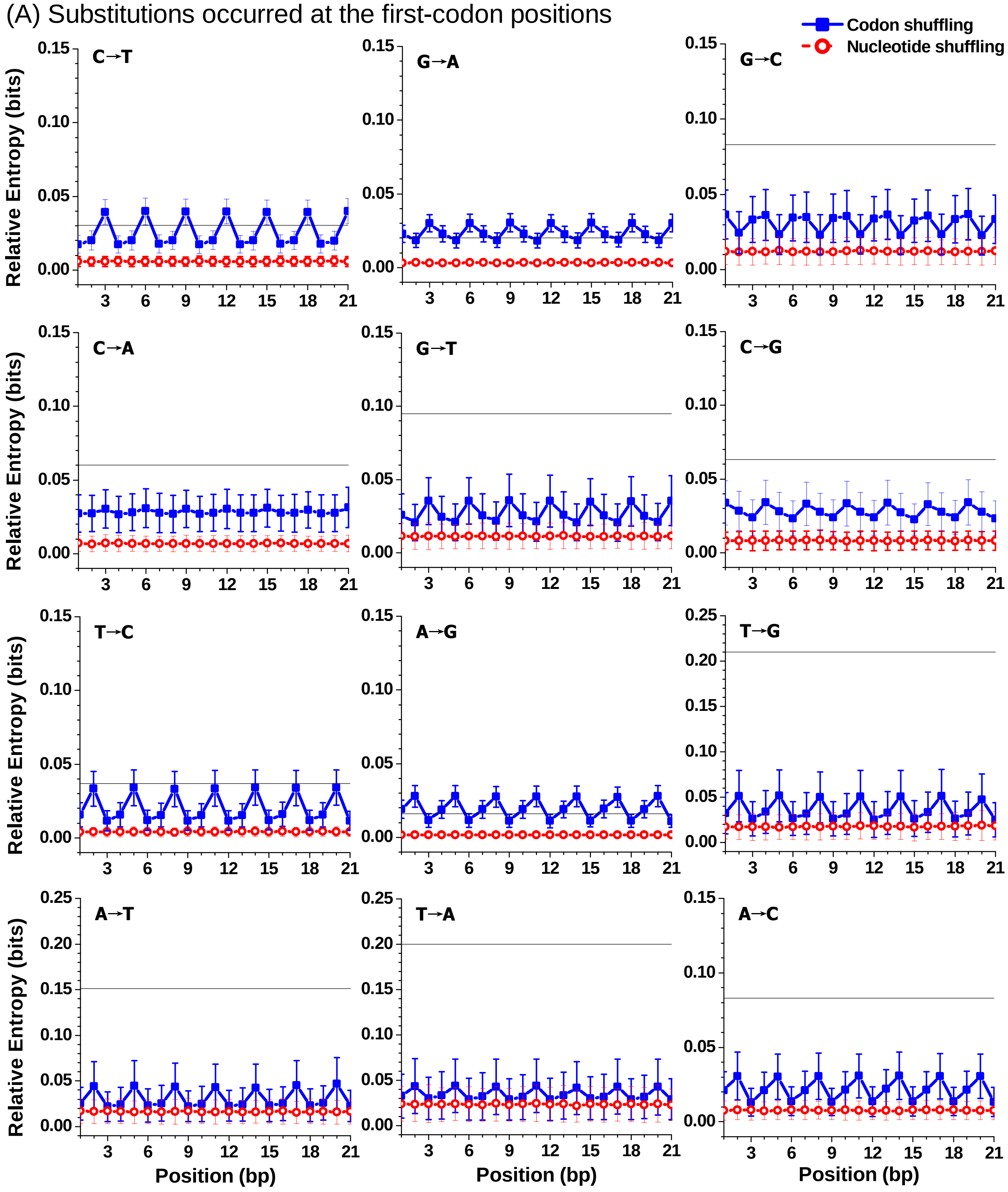

(B) Substitutions occurred at the second-codon positions

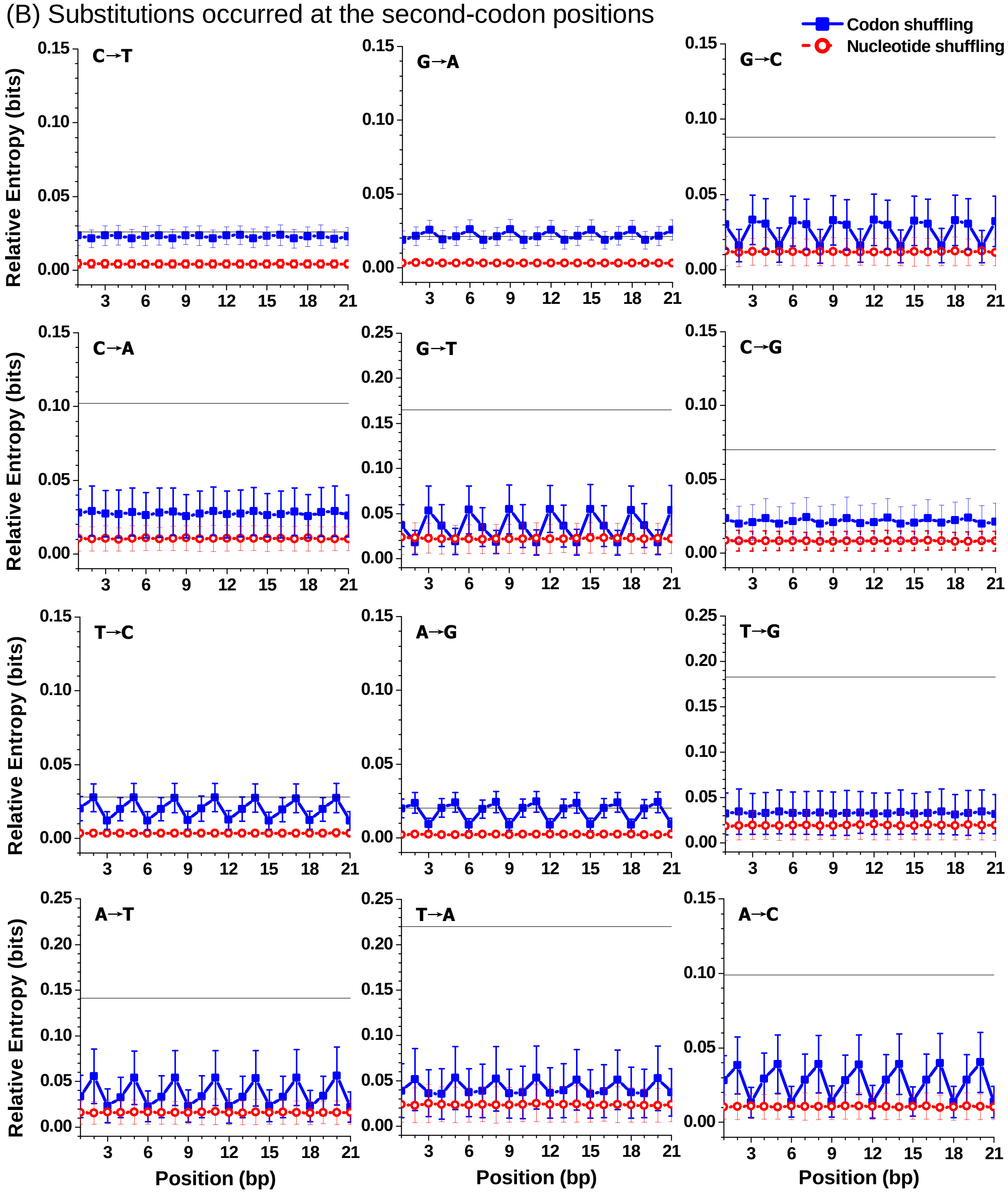

(C) Substitutions occurred at the third-codon positions

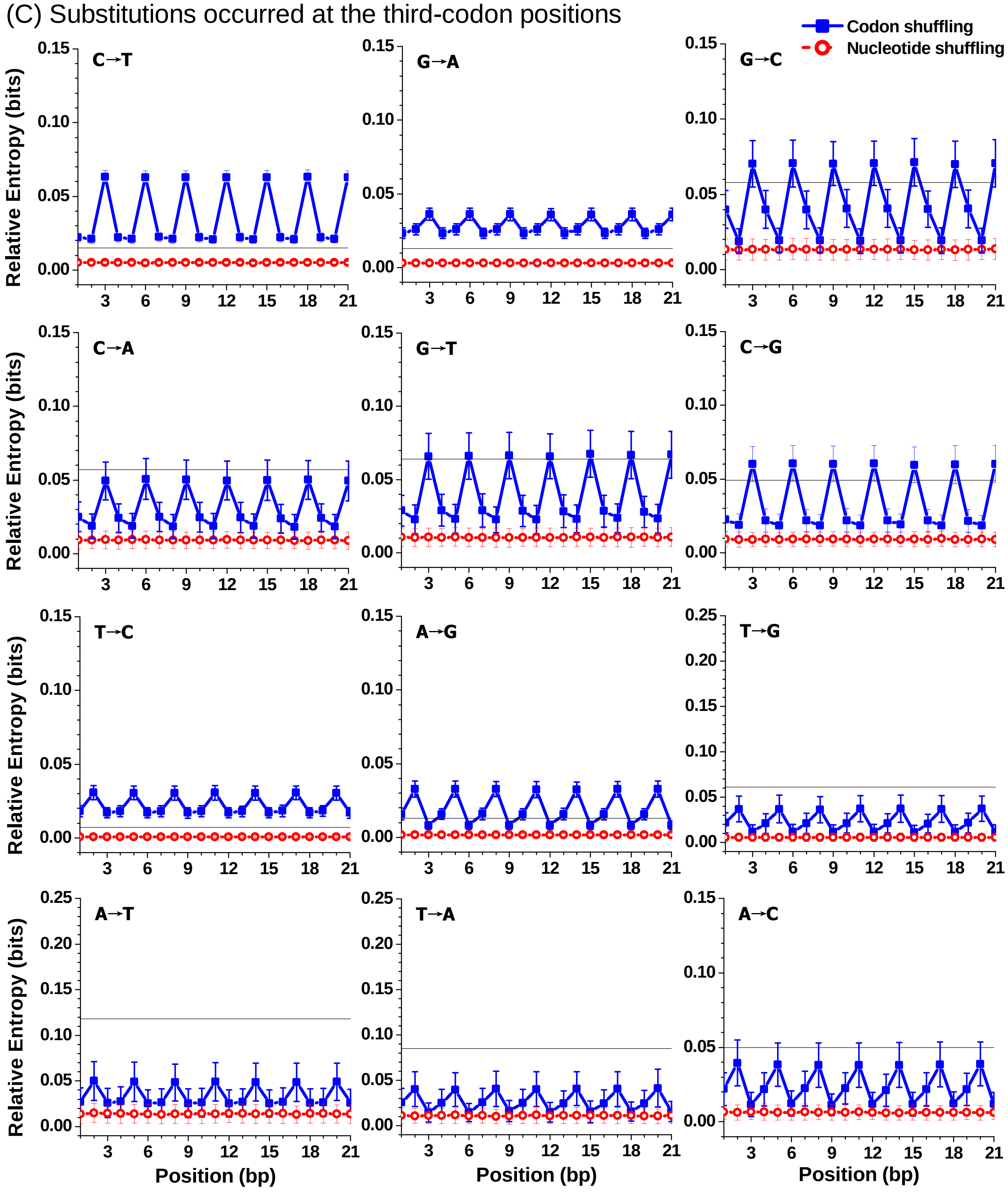

Supplement: Additional file 6 — Relative entropies of the nucleotide- and codon-shuffled datasets. This file illustrates relative entropies across 21 nucleotide positions that were randomly chosen from nucleotide- and codon-shuffled sequences. The figure legend refers to Figure 4 except for labels on the X-axis. Points and error bars in the figures represent means and standard deviations for 1,000 independently random samples. [file 1471-2164-11-416-S6.PDF]
