# Supplementary material for: Patterns of nucleotides that flank substitutions in human orthologous genes
Source: BMC Genomics. 2010 Jul 5;11:416. doi: 10.1186/1471-2164-11-416 (PMC2996944; doi:10.1186/1471-2164-11-416)
Supplement: Additional file 7 — Counterparts of Figure 5: Entropies in native and synonymous-codon-shuffled datasets for substitutions that occurred at the first (A) and second (B) codon positions. This file illustrates relative entropies in the native and synonymous-codon-shuffled dataset for substitutions that occurred at the first (A) and second (B) codon positions. The figure legend refers to Figure 5. [file 1471-2164-11-416-S7.PDF]

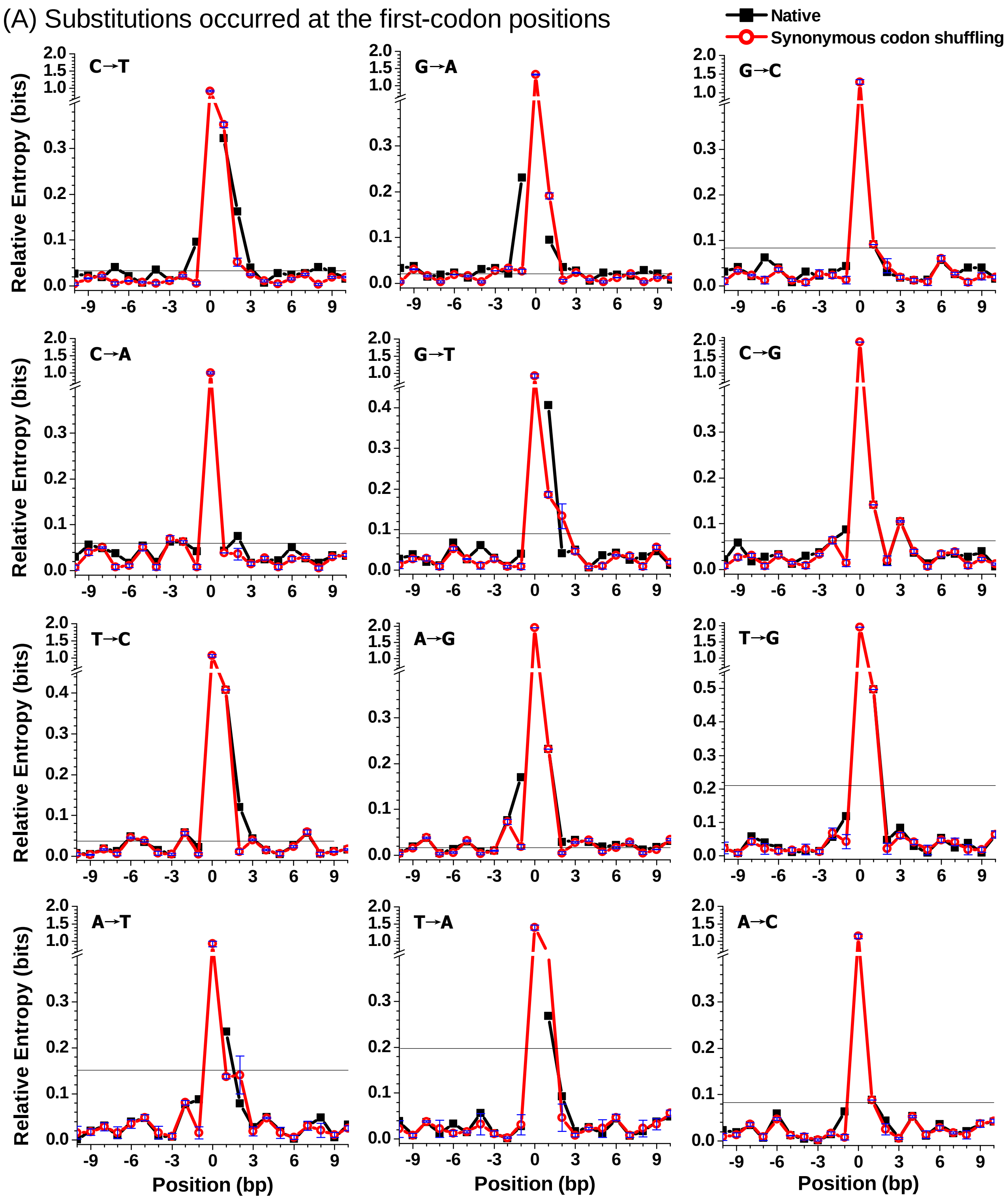
(A) Substitutions occurred at the first-codon positions
Native
Synonymous codon shuffling
C→T
G→A
G→C
C→A
G→T
C→G
T→C
A→G
T→G
A→T
T→A
A→C
Relative Entropy (bits)
Position (bp)

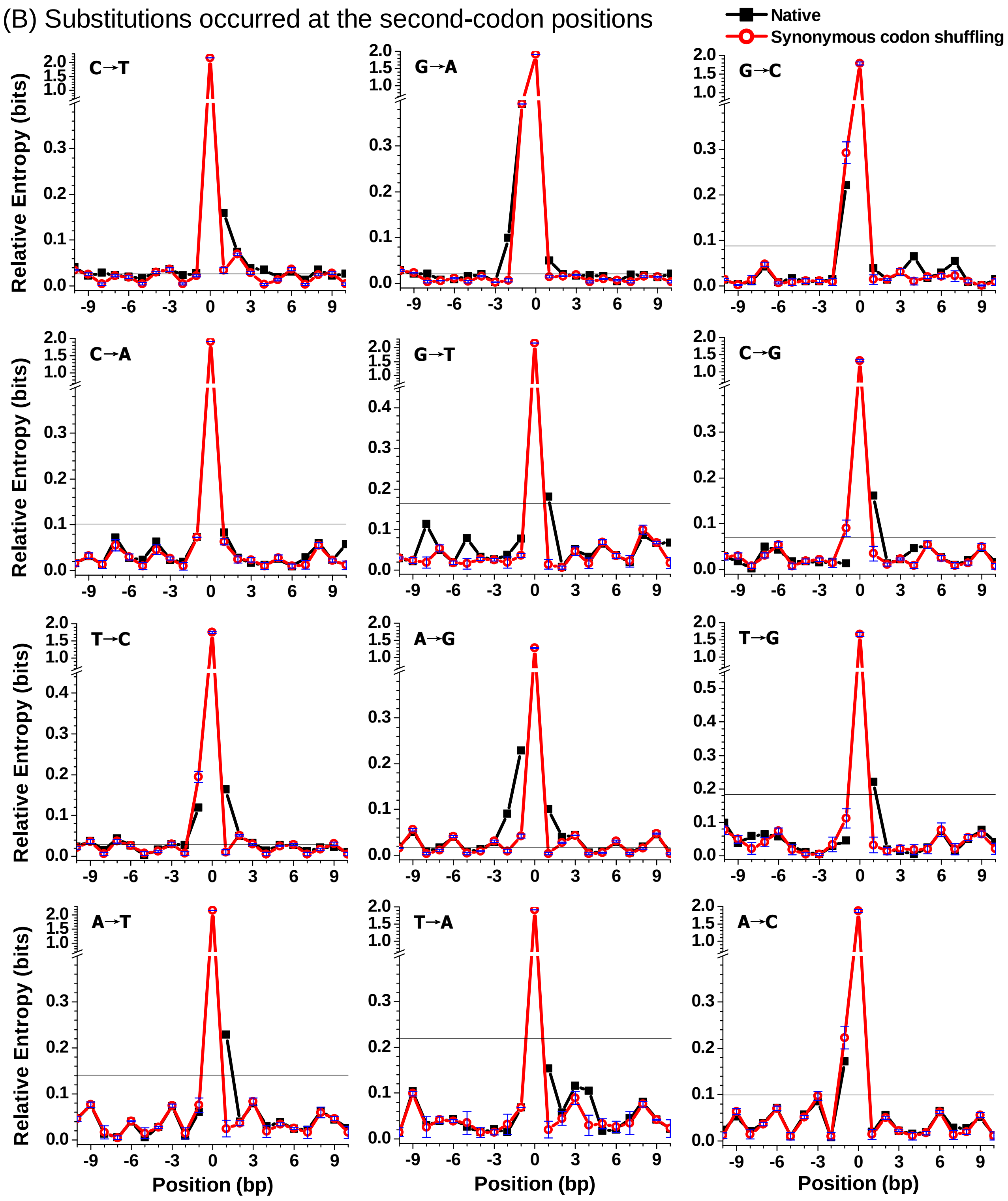
(B) Substitutions occurred at the second-codon positions
Native
Synonymous codon shuffling
C→T
G→A
G→C
C→A
G→T
C→G
T→C
A→G
T→G
A→T
T→A
A→C
Relative Entropy (bits)
Position (bp)
