# Supplementary material for: Patterns of nucleotides that flank substitutions in human orthologous genes
Source: BMC Genomics. 2010 Jul 5;11:416. doi: 10.1186/1471-2164-11-416 (PMC2996944; doi:10.1186/1471-2164-11-416)

Output results from the MCA of SPSS version 16.0

1. Analysis for the relationship among the 3’ (+1) adjacent base, substitution categories and gene GC% groups.

1.1 Model Summary

Model Summary

Dimension	Cronbach's Alpha	Variance Accounted For		
		Total (Eigenvalue)	Inertia	% of Variance
1	.383	1.343	.448	44.774
2	.258	1.207	.402	40.245
Total		2.551	.850	
Mean	.324[a]	1.275	.425	42.510

a. Mean Cronbach's Alpha is based on the mean Eigenvalue.

1.2 Discrimination Measures

Discrimination Measures

	Dimension		Mean
	1	2	
GC%	.504	.083	.293
Substitution	.419	.621	.520
3' adjacent base	.420	.503	.462
Active Total	1.343	1.207	1.275
% of Variance	44.774	40.245	42.510

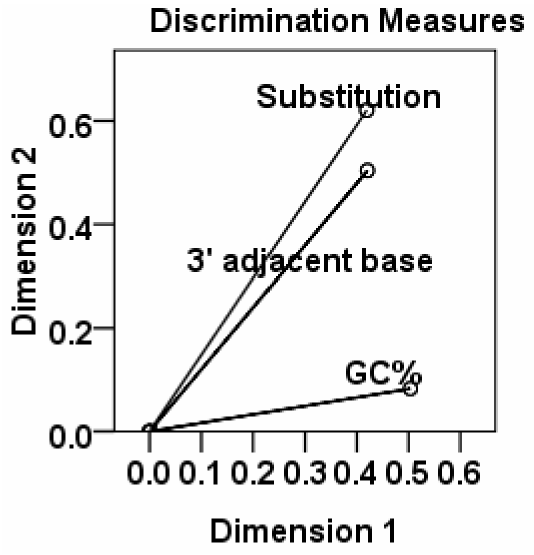

2. Analysis for the relationship among the 5' (-1) adjacent base, substitution categories and gene GC% groups.

2.1 Model Summary

Model Summary

Dimension	Cronbach's Alpha	Variance Accounted For		
		Total (Eigenvalue)	Inertia	% of Variance
1	.345	1.299	.433	43.290
2	.231	1.182	.394	39.406
Total		2.481	.827	
Mean	.291[a]	1.240	.413	41.348

a. Mean Cronbach's Alpha is based on the mean Eigenvalue.

2.2 Discrimination Measures

Discrimination Measures

	Dimension		Mean
	1	2	
GC%	.563	.017	.290
Substitution	.421	.614	.518
5' adjacent base	.314	.551	.432
Active Total	1.299	1.182	1.240
% of Variance	43.290	39.406	41.348

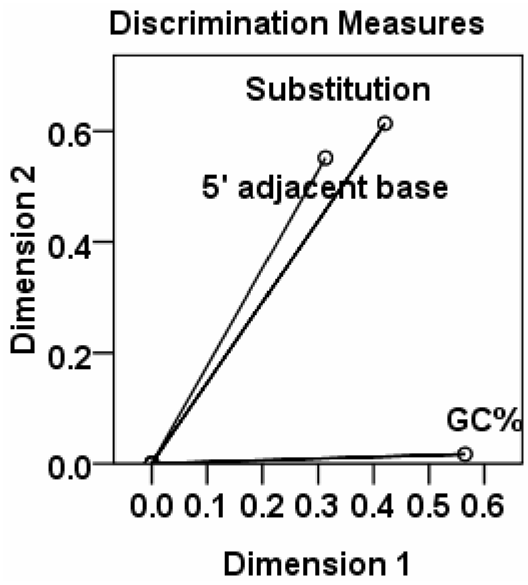

3. Analysis for the relationship among the 3’ (+1) adjacent base, the 5’ (-1) adjacent base, substitution categories and gene GC% groups.

3.1 Model Summary

Model Summary

Dimension	Cronbach's Alpha	Variance Accounted For		
		Total (Eigenvalue)	Inertia	% of Variance
1	.365	1.377	.344	34.415
2	.296	1.285	.321	32.125
Total		2.662	.665	
Mean	.331[a]	1.331	.333	33.270

a. Mean Cronbach's Alpha is based on the mean Eigenvalue.

3.2 Discrimination Measures

Discrimination Measures

	Dimension		Mean
	1	2	
3' adjacent base	.305	.371	.338
5' adjacent base	.139	.287	.213
GC%	.588	.023	.305
Substitution	.345	.604	.475
Active Total	1.377	1.285	1.331
% of Variance	34.415	32.125	33.270

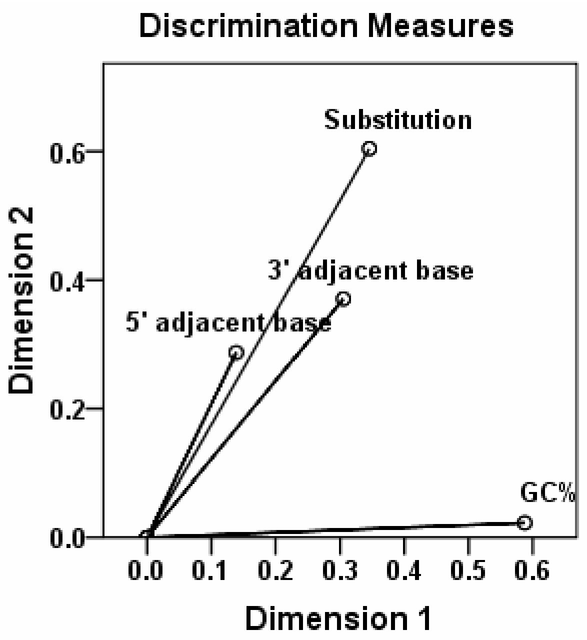

Supplement: Additional file 8 — Output of SPSS MCA. This file illustrates the output results from the MCA of SPSS 16.0. [file 1471-2164-11-416-S8.PDF]
